# Gender Matter, Social Phobia and High-Risk Behaviors in Young Medical Students

**DOI:** 10.4314/ejhs.v31i2.19

**Published:** 2021-03

**Authors:** Naghme Afshari, Jamileh Farokhzadian, Kamel Abdi, Hojjat Sheikhbardsiri

**Affiliations:** 1 Student Research Committee, Kerman University of Medical Sciences, Kerman, Iran; 2 Nursing Research Center, Kerman University of Medical Sciences, Kerman, Iran; 3 Nursing Department, Faculty of Medicine, Komar University of Science and Technology, Sulimaniya, Kurdistan Region, Iraq; 4 Health in Disasters and Emergencies Research Center, Institute for Futures Studies in Health, Kerman University of Medical Sciences, Kerman, Iran

**Keywords:** Gender role, social phobia, risk-taking

## Abstract

**Background:**

Young is one of the most sensitive stages of human life. Social phobia and high-risk behaviors are factors that enhance young crises. This study aimed to determine the relationship between gender role, social phobia and high-risk behaviors among young medical students.

**Methods:**

In this descriptive correlational study, 400 students were selected by quota sampling method from a medical university in Southeastern Iran. For data collection, the demographic information questionnaire, Gender Trait Index (GTI), Social Phobia Inventory (SPIN), and Iranian Adolescent and Young Risk-Taking Scale (IAYRS) were used. Data were analyzed using descriptive statistics including mean and SD and analytic statistics such as Kolmogorov-Smirnov, Mann-Whitney U and Kruskal-Wallis tests using SPSS 25 and p ≤ .05.

**Results:**

The mean scores of masculinity and femininity gender roles were 38.98 ± 7.92 and 44.12 ± 7.76, respectively. Also, 70.5% of the students had dominant feminine traits, and the gender identity was high in 58.8% of the students and moderate in 40.2% of them. Social phobia (37.12 ± 12.61) and high-risk behaviors (81.77 ± 26.08) were moderate. A significant inverse relationship was found between masculine traits and social phobia (p <0.001). Another significant inverse relationship was observed between feminine traits and high-risk behaviors (p <0.05).

**Conclusion:**

Given the poor relationship between gender role, social phobia and high-risk behaviors, it is essential to conduct further studies to determine the predictors of social phobia and high-risk behaviors in medical students.

## Introduction

Young age is associated with rapid growth, physical maturity, social relations, and psychological changes (1). Young age is between 10 and 24 years. Young is a transitional phase of growth and development between childhood and adulthood; it is associated with serious changes in physical, psychological, social, and mental dimensions (2). The World Health Organization defines young age as the occurrence of secondary sex traits, moving towards adulthood, and development of fertility, mental development, identity, the transition from socio-economic dependence to independence (3). Gender role is a social role encompassing a range of behaviors and attitudes that are generally considered acceptable, appropriate, or desirable for people based on their biological or perceived sex. Gender roles are usually centered on conceptions of masculinity and femininity (4). Gender identity is influenced by environmental and biological factors and educational settings are the most important institutions for developing healthy gender identity (5). Gender roles are in the forms of feminine, masculine, androgynous and undifferentiated. According to Bem, every person sees and behaves in the world through his or her lens (6).

Gender role can be effective for the relationships, self-perception, leisure activities, and health. For example, when men choose female-type occupations such as nursing, which is partly accepted as a feminine role by the society, they suffer from anxiety and social phobia as it is incompatible with their gender role (7). Social phobia is anxiety that disturbs the individual's performance in social situations (8). This disorder can be associated with other psychiatric disorders such as high-risk behaviors (9). Ranta et al. reported that 27–47% of youth experienced social phobia in Finland (10). A Canadian study found that 24% of students who dropped out of school had social phobia and high-risk behaviors such as alcohol dependence (11). The results of some studies have demonstrated the association between social phobia and gender with the level of social phobia in female individuals being higher than that of male ones (8,12). Hummadi et al. (2014) found that 44% of university students in Baghdad had social phobia; this figure in girls was 3.5 times higher than that of boys (13). Baloglu et al. in Ankara found that male students experienced social anxiety due to personal problems and entertained themselves by browsing on the internet to forget their problems (14). Another study indicated that the individuals used alcohol, drugs, and psychedelics to reduce fear, increase self-esteem, and achieve better social performance (15).

Gender role seems to be one of the factors affecting social phobia and high-risk behaviors. In other words, male and female genders have differences despite having many biological similarities that should be considered in educational planning Moreover, medical students should reinforce their self-esteem to establish a proper relationship with patients (16). Therefore, it is necessary to investigate the factors affecting social phobia and high-risk behaviors in medical students. Also, the roles defined for women in Iranian culture and religious society differentiate the gender role of Iranian women from those in other countries(9). Therefore, further study is needed to determine the relationship between the gender role of Iranian women and their mental health. Thus, this study aimed to determine the relationship between gender role, social phobia and high-risk behaviors in medical students. More specifically, this study aimed to answer the following research questions: (1) what are the levels of gender role among medical students? (2) What are the levels of social phobia perceived by the medical students? (3) What are the levels of high-risk behaviors in medical students (4) and what are the relationships between gender role, social phobia and high-risk behaviors in medical students?

## Methods

**Design and settings**: This descriptive correlational study was carried out in faculties affiliated to Kerman University of Medical Sciences in Southeastern Iran in 2019.

**Participants and procedures**: The study population was all students of faculties affiliated to Kerman University of Medical Sciences who were selected by quota sampling (N= 3630). The sample size was 400 students using Morgan's table. The inclusion criteria were students aged between 18 and 24 years old with good physical and mental health. Therefore, 110, 41, 66, 41, 73, 61 and 8 students were included from the faculties of medicine, dentistry, pharmacy, nursing and midwifery, allied medicine, health and management, respectively.

**Ethics approval and consent to participate**: The code of ethics (IR.KMU.REC.1397.257) was obtained from the ethics committee of Kerman University of Medical Sciences. To conduct the study, the first researcher presented an introduction letter to make the necessary coordination with the faculty's authorities. Later, written consent forms were obtained from the authorities. Then, the aims of the study and data collection method were described briefly to the eligible participants. Written consent forms were obtained from the participants, and they were ensured about confidentiality and anonymity of the data.

## Data Collection Tools

**Demographic information questionnaire**: It includes gender, age, place of residence, marital status, educational program, field of study, semester, Grade Point Average (GPA), being native or non-native, father's education, mother's education, father's job and mother's job.

**Gender trait index**: Schetzer et al. (2008) designed the index. This scale contains 16 items. Items 1–8 evaluate masculine traits, and items 9–16 evaluate feminine traits. The responses to items are specified by 7-point Likert scale from completely false=1 to completely true = 7. A score between 16 and 48 indicates low gender identity, scores between 48 and 80 indicate moderate gender identity, and a score above 80 indicates high gender identity. Using this test, the individuals obtained scores of masculinity and femininity. The scores of masculinity and feminine scale in bisexual individuals are above the average. A person whose femininity score is high is considered as dominant feminine traits and vice versa. Finally, those who acquire low scores in both scales are known as undifferentiated (17). In the study by Yazdani et al. (2018) in Iran, Cronbach's alpha of this scale was calculated 0.78 and 0.71 for feminine and masculine traits, respectively (18). Cronbach's alpha of the index in the present study was 0.85 and 0.80 for females and males, respectively.

**Social phobia inventory**: Conor et al. (2000) developed the inventory to measure social phobia. This scale contains 17 items and three sub-scales of fear (6 items), avoidance (7 items), and physiology (4 items). The scale scoring is based on 5-point Likert scale (not at all =1, a little bit = 2, somewhat = 3, very much = 4, and extremely = 5). A score of 17–34 indicates weak social phobia, a score of 34–51 indicates moderate social phobia, and a score above 51 indicates severe social phobia (19). Cronbach's alpha coefficient of social phobia inventory in the present study was 0.91.

**Adolescent and Young risk-taking scale**: Zadeh Mohammadi et al. (2011) designed the 38-item scale in Iran to measure the young risktaking in seven dimensions. These dimensions are the tendency to drugs (8 items), the tendency to alcohol (6 items), the tendency to smoking (5 items), the tendency to violence (5 items), the tendency to sexual behavior (4 items), the tendency to the opposite sex (4 items) and the tendency to high-risk driving (6 items). The questionnaire is answered based on the 5-point Likert scale (Strongly disagree = 1 to Strongly agree = 5). A score of 38–76 indicates low young risk-taking; a score of 76–114 indicates moderate young risk-taking; and a score above 114 indicates high young risk-taking (20). The developers of the scale approved the content validity of the questionnaire through the exploratory factor analysis and showed that the dimensions explained 64.84% of the risk-taking variance. Cronbach's alpha was 0.94 for the whole scale and 0.74–0.93 for its subscales(20). Cronbach's alpha for young risk-taking scale was 0.92 in the present work.

**Data analysis**: For data analysis, SPSS software (v.25) was used. The descriptive statistics techniques such as frequency, percentage, mean, and standard deviation were used to describe the characteristics of the samples. Spearman correlation coefficient was used to investigate the relationship between gender role and social phobia and high-risk behaviors. Moreover, given the lack of parametric conditions, Mann-Whitney U and Kruskal-Wallis tests were used to compare the gender role score in terms of demographic variables. Besides, the significance level was set at p<0.05.

## Results

The mean age of the students was 20.47 ± 1.65, and 49.8% (n=199) of them were females. The mean GPA of the students was 16.24 ± 1.82, and 95.5% of them were single. The majority of the students (27.5%) were from the school of Medicine; 54% of the students had professional doctorate/Master's degrees; and the rest were undergraduates. About 70.2% of the students were native, and 98% of the students' parents were literate. Equally, the parents of 39% of students had bachelor's degree. Also, 93.8% of the students were living with both parents, shown in Table 1.

**Table 1:** the relationship between demographic characteristics of the medical students and gender role

Variable	Frequency	%	Feminine traits		Statistical test / P value	masculine traits		Statistical test / P value
	
			Mean	SD		Mean	SD	
**Gender**								
**Female**	199	49.8	45.27	7.52	Z= -2.9	37.66	7.85	Z= -3.05
**Male**	201	50.2	42.98	7.84	P= 0.004	40.29	7.8	P= 0.002
**Marital Status**								
**Single**	382	95.5	44.11	7.59	Z= 1.14	38.89	7.82	Z= -1.87
Married	18	4.5	45.38	10.85	P= 0.26	43	8.13	P= 0.06
**Field of Study**								
**Medicine**	110	27.5	44.57	8.22	H= 5.13	38.96	8.93	H= 3.33
**Dentistry**	41	10.3	43.73	7.79	P= 0.53	38.63	7.09	P= 0.77
**Pharmacy**	66	16.3	44.11	7.5		39.95	7.44	
**Nursing and** **Midwifery**	41	10.3	44.98	7.09		39.39	8.37	
**Allied Medicine**	73	18.1	42.99	7.54		37.96	8.41	
**Public Health**	61	15.5	43.9	8.21		38.9	6.47	
**Management**	8	2	47.62	4.17		41	5.07	
**Educational Program**								
**professional** **/doctorate/** **Master's degrees**	216	54	44.27	7.9	Z= -0.59 P= 0.56	39.2	8.16	Z= -0.45 P= 0.65
**BSc**	184	46	43.94	7.6		38.73	7.66	
**Semester**								
**First**	47	11.8	42.74	7.82	H= 7.45	36.77	7.04	H= 11.13
**Third**	91	22.8	43.1	8.07	P= 0.28	37.67	8.15	P= 0.08
**Fifth**	128	32	44.58	7.93		40.2	7.82	
**Seventh**	89	22.3	44.37	7.54		39.62	8.2	
**Ninth**	19	4.8	47.53	6.1		39.63	6.5	
**Eleventh**	17	4.3	44.05	7.72		39.41	9.27	
**Thirteenth**	9	2.3	45.56	5.52		40.56	6.89	
**Geographical** **location**								
**Native**	281	70.2	44.46	7.88	Z= -1.38	38.69	8.11	Z= -0.8
**Non-native**	119	29.8	43.33	7.43		39.68	7.46	
**Father's Education**								
**Illiterate**	7	1.8	45	4.65	H= 4.84	35.71	8.42	H= 1.54
**No degree**	39	9.8	44.18	9.96	P= 0.44	39.72	8.45	P= 0.91
**High school** **diploma**	94	23.5	44.58	7.17		39.31	7.79	
**BSc**	157	39.2	43.1	7.68		38.59	7.98	
**General Physician** **Master's/**	67	16.7	45.28	7.73		39.07	8.01	
**PhD**	36	9	44.97	7.3		39.53	7.49	
**Mother's Education**								
**Illiterate**	8	2	43.38	5.73	H= 1.52	38.88	7.92	H= 3.5
**No degree**	57	14.2	44.14	9.16	P= 0.91	39.02	7.92	P= 0.62
**High school** **diploma**	119	29.8	44.16	7.35		39.79	8.19	
**BA/BSc**	159	39.7	43.86	7.71		38.89	7.54	
**GP /MSc**	43	10.8	44.95	7.63		38.02	8.65	
**Specialist /PhD**	14	3.5	44.5	7.87		36.07	7.89	
**Father's Job**								
**Self-employed**	115	28.8	44.15	8.25	H= 2.59	39.45	8.05	H= 2.59
**employed**	172	43	43.52	7.9	P= 0.63	38.75	7.74	P= 0.63
**Unemployed**	2	0.5	48.5	10.61		32.5	4.95	
**Retired**	109	27.2	44.97	6.96		39.06	8.13	
**Disabled**	2	0.5	43	5.66		34.5	9.19	
**Mother's Job**								
**Self-employed**	21	5.3	45.43	8.69	H= 1.47	39.43	8.69	H= 8.77
**employed**	120	32.5	44.26	7.94	P= 0.83	37.94	7.64	P= 0.07
**Homemaker**	207	51.7	43.91	7.85		39.75	7.67	
**Retired**	10	10	44	6.4		38.85	9.12	
**Disabled**	5	0.5	45.5	2.12		25.5	3.53	

The mean scores for gender roles of masculinity and femininity were 38.98 ± 7.92 and 44.12 ± 7.76, respectively. The item “assertive” (4.19 ± 1.55) had the lowest score of masculine traits, and the item “competitive” (5.26 ± 1.44) had the highest score of masculine traits. The items “eager to soothe hurt feelings” and “gentle” (5.37 ± 1.42) had the lowest scores of feminine traits, and the item “affectionate” (5.71 ± 1.3) had the highest score of feminine traits, shown in Table 2. Moreover, 70.5% (282 individuals) of the students had dominant female traits, and 23.5% (94 individuals) had dominant male traits. Gender identity was poor in 1% of the students, moderate in 40.2% of them, and high in 58.8% of them.

**Table 2:** mean score of Gender Trait Index items

Item	Mean	SD
Have leadership abilities	5.12	1.4
Assertive	4.19	1.55
Willing to take a stand	4.71	1.59
Ambitious	4.46	1.81
Competitive	5.26	1.44
A strong personality	5.12	1.43
Forceful	5.14	1.33
Act like a leader	4.91	1.49
Total score of masculine traits	38.98	7.92
Affectionate	5.71	1.3
Tender	5.37	1.51
Sensitive to others' needs	5.64	1.26
Sympathetic	5.67	1.19
Warm	5.61	1.3
Eager to soothe hurt feelings	5.37	1.42
Gentle	5.38	1.43
Compassionate	5.40	1.43
Total score of feminine traits	44.12	7.76

The result showed that the mean score of social phobia was at a moderate level (37.12 ± 12.61). Besides, 46.3% of the students had low social phobia; 41.5% of them had moderate social phobia, and 13.2% of them had high social phobia, shown in Table 3. The result showed that the mean score of high-risk behaviors among medical students was at a moderate level (81.77 ± 26.08). Overall, 46.3% of the students had low risk-taking, 40.5% of them had moderate risk-taking, and 13.2% of them had high risk-taking, shown in Table 3. A significant inverse relationship was found between the scores of feminine traits and avoidance dimension. Therefore, the higher the score of feminine traits, the lower is the score of avoidance dimension and vice versa. A significant inverse relationship was found between the scores of masculine traits and social phobia and all of its dimensions. In other words, the higher the score of masculine traits, the lower is the scores of social phobia and its dimensions. Furthermore, a significant inverse relationship was found between the scores of feminine traits and high-risk behaviors and all of its dimensions. In other words, the higher the score of feminine traits, the lower is the high-risk behaviors, and vice versa. A significant direct relationship was found between the scores of masculine traits and dimensions of a tendency to violence, the tendency to sexual behavior and relations, the tendency to relations with the opposite sex and the tendency to high-risk driving. In other words, the higher the score of masculine traits, the higher is the scores of tendencies to violence, the tendency to sexual behaviors and relations, the tendency to relations with the opposite sex and the tendency to high-risk driving, shown in Table 4.

**Table 3:** Mean scores of social phobia and its dimensions as well as high-risk behaviors and its dimensions in medical students

Variable		Mean	SD
**Social** **phobia**	Fear	12.76	4.54
	Avoidance	15.22	5.59
	Physiology	9.14	3.64
	Total social phobia	37.12	12.61
**High-risk behaviors**	Tendency to narcotics	12.94	6.26
	Tendency to alcohol	11.73	6.03
	Tendency to smoking	9.06	5.0
	Tendency to violence	10.43	4.16
	Tendency to sexual behavior and relations	9.3	4.6
	Tendency to relations with the opposite sex	10.93	4.49
	Tendency to high-risk driving	17.41	6.12
	Total high-risk behaviors	81.77	26.08

**Table 4:** relationship between gender role and social phobia and its dimensions as well as high-risk behaviors and its dimensions in the medical students

Variable		Gender role			

		Feminine traits		Masculine traits	
		Spearman correlation coefficient	P value	Spearman correlation coefficient	P value
**Social** **phobia**	Fear	-0.08	0.12	-0.27	<0.001
	Avoidance	-0.10	0.04	-0.32	<0.001
	Physiology	-0.03	0.52	-0.12	0.02
	Total social phobia	-0.09	0.08	-0.27	<0.001
**High-risk behaviors**	Tendency to narcotics	-0.19	<0.001	0.001	0.99
	Tendency to alcohol	-0.16	0.001	0.07	0.17
	Tendency to smoking	-0.11	0.02	0.002	0.97
	Tendency to violence	-0.18	<0.001	0.14	0.01
	Tendency to sexual behaviors and relations	-0.17	0.001	0.17	0.001
	Tendency to relations with the opposite sex	-0.12	0.02	0.08	0.11
	Tendency to high-risk driving	-0.13	0.01	0.11	0.03
	Total high-risk behaviors	-0.19	<0.001	0.11	0..03

A significant direct and weak relationship was found between scores of social phobia and dimensions of a tendency to violence and tendency to smoking. Thus, the higher the score of social phobia, the higher are the scores of a tendency to violence and tendency to smoking, and vice versa, shown in Table 5.

**Table 5:** relationship between the scores of social phobia and high-risk behaviors and its dimensions in the medical students

Variable	Social phobia	

	Spearman correlation coefficient	P value
Tendency to narcotics	0.07	0.18
Tendency to alcohol	0.06	0.28
Tendency to smoking	0.15	<0.001
Tendency to violence	0.12	0.02
Tendency to sexual behaviors and relations	0.04	0.41
Tendency to relations with the opposite sex	-0.03	0.59
Tendency to high-risk driving	-0.04	0.47
Total high-risk behaviors	0.07	0.16

A significant direct and weak relationship was found between age and masculine traits. In other words, the older the age, the higher is the masculine traits (r= 0.11, P= 0.03). A significantly weak and inverse relationship was found between GPA and masculine traits. The higher the GPA, the lower are the masculine traits, and vice versa (r= -0.13, P= 0.04). No significant relationship was found between age, GPA and feminine traits. The mean score of feminine traits in women was higher than that of men (Z= -2.9, P= 0.004), and the mean score of male traits in men was higher than that of women (Z= -3.05, P= 0.002). The score of gender role was not significantly different in terms of other demographic variables (P> 0.05) (Table 1).

A significantly direct and weak relationship was found between age and high-risk behaviors. Thus, the older the age, the greater the high-risk behaviors, and vice versa (r= 0.19, P <0.001). Also, a significant weak and inverse relationship was observed between GPA and high-risk behaviors, that is, the higher the GPA, the lower the high-risk behaviors, and vice versa (r= -0.21, P= 0.001). The male students had significantly more high-risk behaviors than female students (Z= -8.26, P <0.001). Besides, the Bonferroni's post-hoc test showed that ninth-semester students had significantly more high-risk behaviors than the first-semester and (P= 0.04) and the fifth-semester ones (P= 0.01). The highrisk behaviors were not significantly different in terms of other demographic characteristics (P> 0.05).

## Discussion

The results of this research showed that the majority of the studied students had dominant female traits, and the gender identity of almost all samples was moderate to high. Noorani et al. (2015) found a significant positive correlation between femininity and masculinity (21). Therefore, those with masculine gender roles are often object-oriented and choose technical jobs such as laboring and engineering, and those with feminine gender roles choose jobs such as medicine, nursing, and teaching. It can be concluded that medical students' field of study is compatible with their gender self-concept. Chan et al. (2012) showed that television programs and advertisements can shape gender roles and masculine or feminine behaviors in individuals (22).

The results of the present study also showed that the social phobia of medical students was moderate and 13.2% of them experienced high social phobia. Amrai et al. (2011) supported the present study and showed that 50% of students with no academic success suffered from anxiety disorders and were unable to speak in front of others since they had functional anxiety (23). Alkhalifah et al. (2017) in Saudi Arabia reported that the mean social phobia in youth students was 37.95, Also 29% of the students had mild social phobia, and 19.8% of them experienced very high social phobia (24). The large sample size in this research and the cultural differences of the statistical populations in the two studies might have led to different results.

In the present study, the mean score of high-risk behaviors among medical students was moderate, and 13.2% of them had high risktaking behavior. Marzban et al. (2018) also conducted a research on students and showed that the mean score of high-risk behaviors among students was moderate 56.25 (25). Ghonchepour et al. (2019) concluded that the mean score of high-risk behaviors in the students from three different universities was moderate. However, the level of risk-taking in medical students was lower than that among students in humanities and non-profit universities (26). The tendency to high-risk behaviors was associated with factors such as young individuals' mental health, religious beliefs, marital status, residence, educational background, type of university and lack of awareness of high-risk behaviors this idea requires further investigation to detect factors reducing high-risk behaviors.

According to our results, a weak relationship between masculine traits and social phobia was found. Thus, those with masculine traits experienced less social phobia than those with feminine traits. Therefore, the higher the masculine traits, the lower is the score of social phobia and its dimensions, and vice versa. A weak relationship was also found between the feminine traits and avoidance dimension. Aliakbari et al. (2017) emphasized that the gender role was effective in social phobia. In this study, the mean score of social phobia in women was higher than that of men (27). However, one study demonstrated no significant relationship between gender and social phobia (28). Due to the weak relationship in the present study between the discussed variables, such a relationship could be due to the large sample size; hence, further research is needed to investigate this matter.

The results of the present research showed a weak relationship between the feminine traits and the score of high-risk behaviors and all its dimensions. Thus, the higher the score of feminine traits, the lower the high-risk behaviors in all dimensions, and vice versa. A weak relationship was found between the scores of masculine traits and the tendency to violence, the tendency to sexual behavior and relations, the tendency to relations with the opposite sex, and the tendency to high-risk driving. Therefore, the higher the score of masculine traits, the higher the scores of the tendency to violence, the tendency to sexual behavior and relations, the tendency to relations with the opposite sex, the tendency to high-risk driving, and the tendency to smoking. Maziak et al. in Syria showed that 47% and 9% of men and women were smoking, respectively, and these studies indicated that although women had high-risk behaviors, they performed fewer high-risk behaviors than men (29).

The results of the present study showed a weak relationship between social phobia and tendency to high-risk behaviors. In other words, the higher the score of social phobia, the higher the scores of the tendency to violence and tendency to smoking, and vice versa. Fox (2011) have found more high-risk behaviors in stressful conditions; anxiety makes a person susceptible to high-risk decision-making due to aggression and impulsivity (30). Kazemi et al. (2017) believed that anxiety and stress before tests were significantly associated with a tendency to highrisk behaviors (31). Since a significantly weak relationship was found between social phobia and tendency to high-risk behaviors in the present study, Fox, and Kazemi (30,31) did not support the present work. The sampling method and various cultural differences might be the reasons for this contradiction.

In the present research, a significant direct and weak relationship was found between age and masculine traits. Thus, the older the age, the higher is the masculine traits. A significant weak and inverse relationship was also found between GPA and masculine traits. Thus, the higher the GPA, the lower is the masculine traits. Also, the mean score of feminine traits in women was higher than that of men, and the mean score of masculine traits in men was higher than that of women. Aliakbari et al. (2014) showed no significant relationship between age and gender roles. Therefore, their study supported the present study (32). However, Forutan et al. (2019) showed that the lower the economic and cultural status of individuals, the higher the tendency to show masculine gender roles and the more preferred the masculine behaviors would be. In contrast, modern society prefers and institutionalizes the feminine gender role (33). Their study was carried out on a large sample in several different provinces that could lead to different results between the two studies.

According to the results of the present study, the students with better educational status showed less high-risk behaviors, and those who were in the final years of young showed more high-risk behaviors. Study of Abbasi supported the present study in which factors such as education level, hormonal changes at various ages, religious status, religious beliefs, type of university, knowledge of life skills, and awareness affect high-risk behaviors (34). The male students also had more high-risk behaviors than the female ones. Dumas (2012) also reported that gender was significantly associated with high-risk behaviors which were more obvious in boys than in girls (35). Given a weak relationship between masculine traits and the score of high-risk behaviors in the present study, these studies supported the present study. Nduna et al. (2010) studied the individuals with feminine and masculine traits in Southern Africa and concluded that fewer men (16%) preferred safe sexual behaviors than women (35%), indicating men's more high-risk behaviors than women (36). The students in the 9^th^ semester had more high-risk behaviors than those of the 1^st^ and 5^th^ semesters. However, Samimi (2016) showed that those with higher academic level had more high-risk behaviors such as sexual behaviors and smoking (37). The contradiction between these two studies is due to the differences in the statistical population, sample size, and data collection methods between the two studies.

The results of the present study showed that two-thirds of the studied students had dominant feminine traits. However, the gender identity of almost all students was moderate to high. The results of the present study also demonstrated that social phobia and high-risk behaviors of medical students were moderate, and less than one-seventh of them had experienced high social phobia and high-risk behaviors. A weak relationship was found between gender role and high-risk behaviors.

This study had several limitations. It was conducted only on the students of Kerman University of Medical Sciences; the results cannot be generalized to other students. A questionnaire was used to estimate the high-risk behaviors. The individuals might have not reported their experience in this questionnaire.

This study has provided an insight into the effect of gender role on social phobia and high-risk behaviors in medical student. The results of the present study can be used as a basis for policy-making in the field of Mental Health promotion education by the ministry of health and medical education in Iran. In educational environments need to that noted to early prevention for prohibit of some high-risk behaviors in students, therefore for access to this purpose, education of students and teachers are necessary. Also, further research on high-risk behaviors and social phobia in youth should be conducted to strengthen and broaden these findings.
